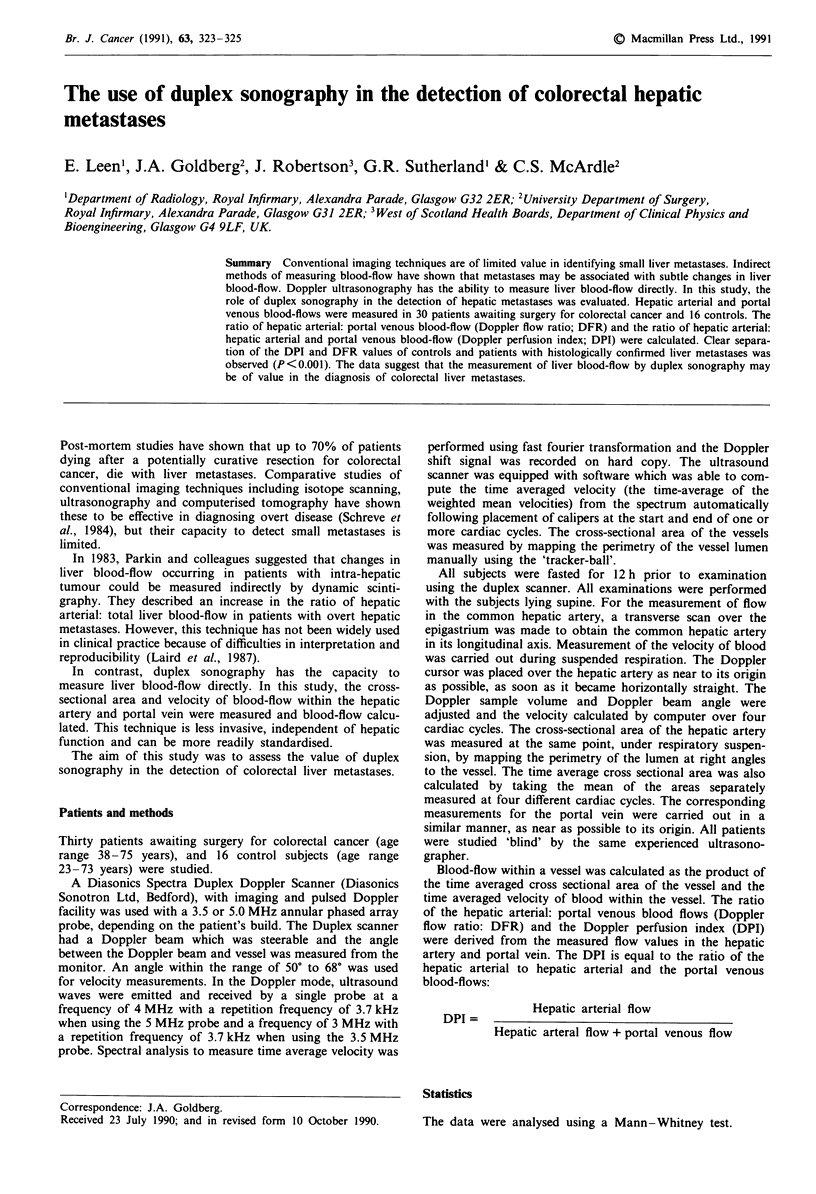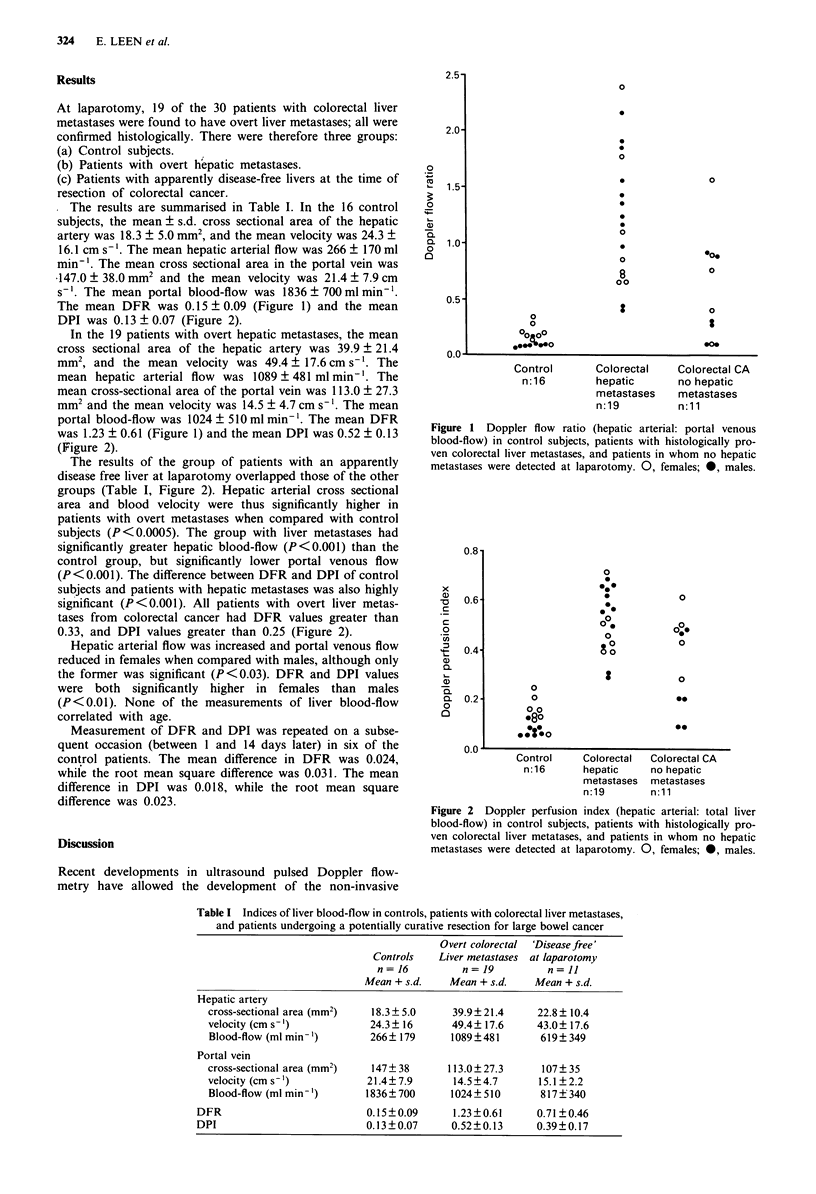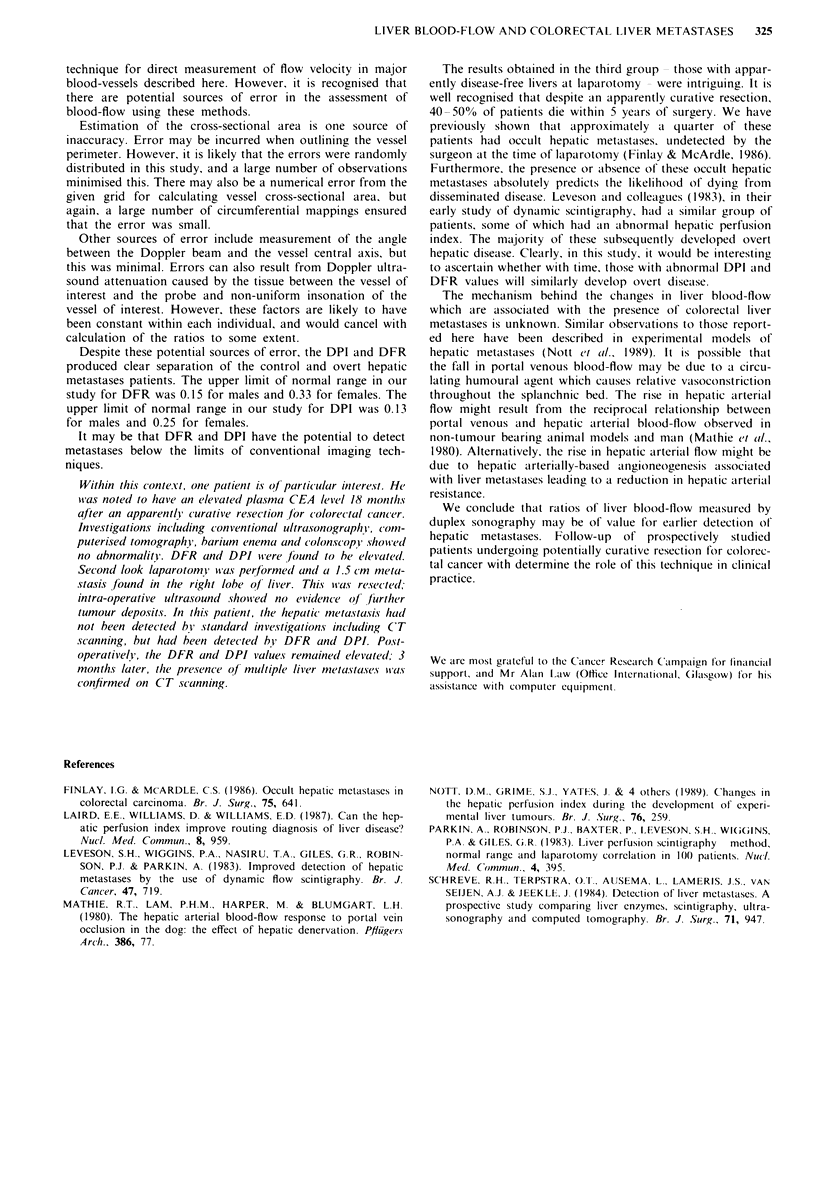# The use of duplex sonography in the detection of colorectal hepatic metastases.

**DOI:** 10.1038/bjc.1991.76

**Published:** 1991-02

**Authors:** E. Leen, J. A. Goldberg, J. Robertson, G. R. Sutherland, C. S. McArdle

**Affiliations:** Department of Radiology, Royal Infirmary, Glasgow, UK.

## Abstract

Conventional imaging techniques are of limited value in identifying small liver metastases. Indirect methods of measuring blood-flow have shown that metastases may be associated with subtle changes in liver blood-flow. Doppler ultrasonography has the ability to measure liver blood-flow directly. In this study, the role of duplex sonography in the detection of hepatic metastases was evaluated. Hepatic arterial and portal venous blood-flows were measured in 30 patients awaiting surgery for colorectal cancer and 16 controls. The ratio of hepatic arterial: portal venous blood-flow (Doppler flow ratio; DFR) and the ratio of hepatic arterial:hepatic arterial and portal venous blood-flow (Doppler perfusion index; DPI) were calculated. Clear separation of the DPI and DFR values of controls and patients with histologically confirmed liver metastases was observed (P less than 0.001). The data suggest that the measurement of liver blood-flow by duplex sonography may be of value in the diagnosis of colorectal liver metastases.


Br. J. Cancer (1991), 63, 323-325 © Macmillan Press Ltd., 1991

### The use of duplex sonography in the detection of colorectal hepatic metastases

E. Leen[1], J.A. Goldberg[2], J. Robertson[3], G.R. Sutherland[1] & C.S. McArdle[2]

[1]*Department of Radiology, Royal Infirmary, Alexandra Parade, Glasgow G32 2ER;* [2]*University Department of Surgery, Royal Infirmary, Alexandra Parade, Glasgow G31 2ER;* [3]*West of Scotland Health Boards, Department of Clinical Physics and Bioengineering, Glasgow G4 9LF, UK.*

**Summary** Conventional imaging techniques are of limited value in identifying small liver metastases. Indirect methods of measuring blood-flow have shown that metastases may be associated with subtle changes in liver blood-flow. Doppler ultrasonography has the ability to measure liver blood-flow directly. In this study, the role of duplex sonography in the detection of hepatic metastases was evaluated. Hepatic arterial and portal venous blood-flows were measured in 30 patients awaiting surgery for colorectal cancer and 16 controls. The ratio of hepatic arterial: portal venous blood-flow (Doppler flow ratio; DFR) and the ratio of hepatic arterial: hepatic arterial and portal venous blood-flow (Doppler perfusion index; DPI) were calculated. Clear separation of the DPI and DFR values of controls and patients with histologically confirmed liver metastases was observed ($P < 0.001$). The data suggest that the measurement of liver blood-flow by duplex sonography may be of value in the diagnosis of colorectal liver metastases.

Post-mortem studies have shown that up to 70% of patients dying after a potentially curative resection for colorectal cancer, die with liver metastases. Comparative studies of conventional imaging techniques including isotope scanning, ultrasonography and computerised tomography have shown these to be effective in diagnosing overt disease (Schreve *et al.*, 1984), but their capacity to detect small metastases is limited.

In 1983, Parkin and colleagues suggested that changes in liver blood-flow occurring in patients with intra-hepatic tumour could be measured indirectly by dynamic scintigraphy. They described an increase in the ratio of hepatic arterial: total liver blood-flow in patients with overt hepatic metastases. However, this technique has not been widely used in clinical practice because of difficulties in interpretation and reproducibility (Laird *et al.*, 1987).

In contrast, duplex sonography has the capacity to measure liver blood-flow directly. In this study, the cross-sectional area and velocity of blood-flow within the hepatic artery and portal vein were measured and blood-flow calculated. This technique is less invasive, independent of hepatic function and can be more readily standardised.

The aim of this study was to assess the value of duplex sonography in the detection of colorectal liver metastases.

### Patients and methods

Thirty patients awaiting surgery for colorectal cancer (age range 38–75 years), and 16 control subjects (age range 23–73 years) were studied.

A Diasonics Spectra Duplex Doppler Scanner (Diasonics Sonotron Ltd, Bedford), with imaging and pulsed Doppler facility was used with a 3.5 or 5.0 MHz annular phased array probe, depending on the patient's build. The Duplex scanner had a Doppler beam which was steerable and the angle between the Doppler beam and vessel was measured from the monitor. An angle within the range of 50° to 68° was used for velocity measurements. In the Doppler mode, ultrasound waves were emitted and received by a single probe at a frequency of 4 MHz with a repetition frequency of 3.7 kHz when using the 5 MHz probe and a frequency of 3 MHz with a repetition frequency of 3.7 kHz when using the 3.5 MHz probe. Spectral analysis to measure time average velocity was performed using fast fourier transformation and the Doppler shift signal was recorded on hard copy. The ultrasound scanner was equipped with software which was able to compute the time averaged velocity (the time-average of the weighted mean velocities) from the spectrum automatically following placement of calipers at the start and end of one or more cardiac cycles. The cross-sectional area of the vessels was measured by mapping the perimetry of the vessel lumen manually using the 'tracker-ball'.

All subjects were fasted for 12 h prior to examination using the duplex scanner. All examinations were performed with the subjects lying supine. For the measurement of flow in the common hepatic artery, a transverse scan over the epigastrium was made to obtain the common hepatic artery in its longitudinal axis. Measurement of the velocity of blood was carried out during suspended respiration. The Doppler cursor was placed over the hepatic artery as near to its origin as possible, as soon as it became horizontally straight. The Doppler sample volume and Doppler beam angle were adjusted and the velocity calculated by computer over four cardiac cycles. The cross-sectional area of the hepatic artery was measured at the same point, under respiratory suspension, by mapping the perimetry of the lumen at right angles to the vessel. The time average cross sectional area was also calculated by taking the mean of the areas separately measured at four different cardiac cycles. The corresponding measurements for the portal vein were carried out in a similar manner, as near as possible to its origin. All patients were studied 'blind' by the same experienced ultrasonographer.

Blood-flow within a vessel was calculated as the product of the time averaged cross sectional area of the vessel and the time averaged velocity of blood within the vessel. The ratio of the hepatic arterial: portal venous blood flows (Doppler flow ratio: DFR) and the Doppler perfusion index (DPI) were derived from the measured flow values in the hepatic artery and portal vein. The DPI is equal to the ratio of the hepatic arterial to hepatic arterial and the portal venous blood-flows:

$$\text{DPI} = \frac{\text{Hepatic arterial flow}}{\text{Hepatic arteral flow} + \text{portal venous flow}}$$

### Statistics

The data were analysed using a Mann–Whitney test.

Correspondence: J.A. Goldberg.
Received 23 July 1990; and in revised form 10 October 1990.

### Results

At laparotomy, 19 of the 30 patients with colorectal liver metastases were found to have overt liver metastases; all were confirmed histologically. There were therefore three groups:
(a) Control subjects.
(b) Patients with overt hepatic metastases.
(c) Patients with apparently disease-free livers at the time of resection of colorectal cancer.

The results are summarised in Table I. In the 16 control subjects, the mean ± s.d. cross sectional area of the hepatic artery was 18.3 ± 5.0 $mm^2$, and the mean velocity was 24.3 ± 16.1 cm $s^{-1}$. The mean hepatic arterial flow was 266 ± 170 ml $min^{-1}$. The mean cross sectional area in the portal vein was 147.0 ± 38.0 $mm^2$ and the mean velocity was 21.4 ± 7.9 cm $s^{-1}$. The mean portal blood-flow was 1836 ± 700 ml $min^{-1}$. The mean DFR was 0.15 ± 0.09 (Figure 1) and the mean DPI was 0.13 ± 0.07 (Figure 2).

In the 19 patients with overt hepatic metastases, the mean cross sectional area of the hepatic artery was 39.9 ± 21.4 $mm^2$, and the mean velocity was 49.4 ± 17.6 cm $s^{-1}$. The mean hepatic arterial flow was 1089 ± 481 ml $min^{-1}$. The mean cross-sectional area of the portal vein was 113.0 ± 27.3 $mm^2$ and the mean velocity was 14.5 ± 4.7 cm $s^{-1}$. The mean portal blood-flow was 1024 ± 510 ml $min^{-1}$. The mean DFR was 1.23 ± 0.61 (Figure 1) and the mean DPI was 0.52 ± 0.13 (Figure 2).

The results of the group of patients with an apparently disease free liver at laparotomy overlapped those of the other groups (Table I, Figure 2). Hepatic arterial cross sectional area and blood velocity were thus significantly higher in patients with overt metastases when compared with control subjects ($P < 0.0005$). The group with liver metastases had significantly greater hepatic blood-flow ($P < 0.001$) than the control group, but significantly lower portal venous flow ($P < 0.001$). The difference between DFR and DPI of control subjects and patients with hepatic metastases was also highly significant ($P < 0.001$). All patients with overt liver metastases from colorectal cancer had DFR values greater than 0.33, and DPI values greater than 0.25 (Figure 2).

Hepatic arterial flow was increased and portal venous flow reduced in females when compared with males, although only the former was significant ($P < 0.03$). DFR and DPI values were both significantly higher in females than males ($P < 0.01$). None of the measurements of liver blood-flow correlated with age.

Measurement of DFR and DPI was repeated on a subsequent occasion (between 1 and 14 days later) in six of the control patients. The mean difference in DFR was 0.024, while the root mean square difference was 0.031. The mean difference in DPI was 0.018, while the root mean square difference was 0.023.

### Discussion

Recent developments in ultrasound pulsed Doppler flowmetry have allowed the development of the non-invasive

**Figure 1** Doppler flow ratio (hepatic arterial: portal venous blood-flow) in control subjects, patients with histologically proven colorectal liver metastases, and patients in whom no hepatic metastases were detected at laparotomy. ○, females; ●, males.

**Figure 2** Doppler perfusion index (hepatic arterial: total liver blood-flow) in control subjects, patients with histologically proven colorectal liver metatases, and patients in whom no hepatic metastases were detected at laparotomy. ○, females; ●, males.

**Table I** Indices of liver blood-flow in controls, patients with colorectal liver metastases, and patients undergoing a potentially curative resection for large bowel cancer

| | *Controls* *n = 16* *Mean + s.d.* | *Overt colorectal Liver metastases* *n = 19* *Mean + s.d.* | *'Disease free' at laparotomy* *n = 11* *Mean + s.d.* |
|---|---|---|---|
| Hepatic artery | | | |
| cross-sectional area ($mm^2$) | 18.3±5.0 | 39.9±21.4 | 22.8±10.4 |
| velocity (cm $s^{-1}$) | 24.3±16 | 49.4±17.6 | 43.0±17.6 |
| Blood-flow (ml $min^{-1}$) | 266±179 | 1089±481 | 619±349 |
| Portal vein | | | |
| cross-sectional area ($mm^2$) | 147±38 | 113.0±27.3 | 107±35 |
| velocity (cm $s^{-1}$) | 21.4±7.9 | 14.5±4.7 | 15.1±2.2 |
| Blood-flow (ml $min^{-1}$) | 1836±700 | 1024±510 | 817±340 |
| DFR | 0.15±0.09 | 1.23±0.61 | 0.71±0.46 |
| DPI | 0.13±0.07 | 0.52±0.13 | 0.39±0.17 |

technique for direct measurement of flow velocity in major blood-vessels described here. However, it is recognised that there are potential sources of error in the assessment of blood-flow using these methods.

Estimation of the cross-sectional area is one source of inaccuracy. Error may be incurred when outlining the vessel perimeter. However, it is likely that the errors were randomly distributed in this study, and a large number of observations minimised this. There may also be a numerical error from the given grid for calculating vessel cross-sectional area, but again, a large number of circumferential mappings ensured that the error was small.

Other sources of error include measurement of the angle between the Doppler beam and the vessel central axis, but this was minimal. Errors can also result from Doppler ultrasound attenuation caused by the tissue between the vessel of interest and the probe and non-uniform insonation of the vessel of interest. However, these factors are likely to have been constant within each individual, and would cancel with calculation of the ratios to some extent.

Despite these potential sources of error, the DPI and DFR produced clear separation of the control and overt hepatic metastases patients. The upper limit of normal range in our study for DFR was 0.15 for males and 0.33 for females. The upper limit of normal range in our study for DPI was 0.13 for males and 0.25 for females.

It may be that DFR and DPI have the potential to detect metastases below the limits of conventional imaging techniques.

*Within this context, one patient is of particular interest. He was noted to have an elevated plasma CEA level 18 months after an apparently curative resection for colorectal cancer. Investigations including conventional ultrasonography, computerised tomography, barium enema and colonscopy showed no abnormality. DFR and DPI were found to be elevated. Second look laparotomy was performed and a 1.5 cm metastasis found in the right lobe of liver. This was resected; intra-operative ultrasound showed no evidence of further tumour deposits. In this patient, the hepatic metastasis had not been detected by standard investigations including CT scanning, but had been detected by DFR and DPI. Postoperatively, the DFR and DPI values remained elevated; 3 months later, the presence of multiple liver metastases was confirmed on CT scanning.*

The results obtained in the third group – those with apparently disease-free livers at laparotomy – were intriguing. It is well recognised that despite an apparently curative resection, 40–50% of patients die within 5 years of surgery. We have previously shown that approximately a quarter of these patients had occult hepatic metastases, undetected by the surgeon at the time of laparotomy (Finlay & McArdle, 1986). Furthermore, the presence or absence of these occult hepatic metastases absolutely predicts the likelihood of dying from disseminated disease. Leveson and colleagues (1983), in their early study of dynamic scintigraphy, had a similar group of patients, some of which had an abnormal hepatic perfusion index. The majority of these subsequently developed overt hepatic disease. Clearly, in this study, it would be interesting to ascertain whether with time, those with abnormal DPI and DFR values will similarly develop overt disease.

The mechanism behind the changes in liver blood-flow which are associated with the presence of colorectal liver metastases is unknown. Similar observations to those reported here have been described in experimental models of hepatic metastases (Nott *et al.*, 1989). It is possible that the fall in portal venous blood-flow may be due to a circulating humoural agent which causes relative vasoconstriction throughout the splanchnic bed. The rise in hepatic arterial flow might result from the reciprocal relationship between portal venous and hepatic arterial blood-flow observed in non-tumour bearing animal models and man (Mathie *et al.*, 1980). Alternatively, the rise in hepatic arterial flow might be due to hepatic arterially-based angioneogenesis associated with liver metastases leading to a reduction in hepatic arterial resistance.

We conclude that ratios of liver blood-flow measured by duplex sonography may be of value for earlier detection of hepatic metastases. Follow-up of prospectively studied patients undergoing potentially curative resection for colorectal cancer with determine the role of this technique in clinical practice.

We are most grateful to the Cancer Research Campaign for financial support, and Mr Alan Law (Office International, Glasgow) for his assistance with computer equipment.

### References

FINLAY, I.G. & MCARDLE, C.S. (1986). Occult hepatic metastases in colorectal carcinoma. *Br. J. Surg.*, **75**, 641.

LAIRD, E.E., WILLIAMS, D. & WILLIAMS, E.D. (1987). Can the hepatic perfusion index improve routing diagnosis of liver disease? *Nucl. Med. Commun.*, **8**, 959.

LEVESON, S.H., WIGGINS, P.A., NASIRU, T.A., GILES, G.R., ROBINSON, P.J. & PARKIN, A. (1983). Improved detection of hepatic metastases by the use of dynamic flow scintigraphy. *Br. J. Cancer*, **47**, 719.

MATHIE, R.T., LAM, P.H.M., HARPER, M. & BLUMGART, L.H. (1980). The hepatic arterial blood-flow response to portal vein occlusion in the dog: the effect of hepatic denervation. *Pflügers Arch.*, **386**, 77.

NOTT, D.M., GRIME, S.J., YATES, J. & 4 others (1989). Changes in the hepatic perfusion index during the development of experimental liver tumours. *Br. J. Surg.*, **76**, 259.

PARKIN, A., ROBINSON, P.J., BAXTER, P., LEVESON, S.H., WIGGINS, P.A. & GILES, G.R. (1983). Liver perfusion scintigraphy – method, normal range and laparotomy correlation in 100 patients. *Nucl. Med. Commun.*, **4**, 395.

SCHREVE, R.H., TERPSTRA, O.T., AUSEMA, L., LAMERIS, J.S., VAN SEIJEN, A.J. & JEEKLE, J. (1984). Detection of liver metastases. A prospective study comparing liver enzymes, scintigraphy, ultrasonography and computed tomography. *Br. J. Surg.*, **71**, 947.